# Morphology of the Male Reproductive System of the Social Wasp, *Polistes Versicolor Versicolor*, with Phylogenetic Implications

**DOI:** 10.1673/031.010.7101

**Published:** 2010-06-22

**Authors:** Vinícius Albano Araújo, Jane Moreira, José Lino-Neto

**Affiliations:** ^1^ Programa de Pós-Graduação em Entomologia, Department of Animal Biology, Federal University of Viçosa, Minas Gerais, CEP, 36570-000, Brazil; ^2^ Department of General Biology, Federal University of Viçosa, Minas Gerais, CEP, 36570-000, Brazil

**Keywords:** histology, mitotic cycles, spermatozoa, Vespidae, Polistini

## Abstract

Variation in the morphology of the adult male reproductive system among different groups of Hymenoptera offer characteristics that help studies of behavior and the evolutionary history of this group. The objective of this study was to describe the adult male reproductive system of the wasp *Polistes versicolor versicolor* Olivier (Vespidae: Polistini). The reproductive systems were dissected, fixed and embedded for light microscopy. In *P. v. versicolor*, the reproductive system includes a pair of testes, each one with three fusiform follicles. From each follicle emerges an efferent duct that later join together, forming a deferent duct. The first half of the deferent duct is enlarged and differentiated into a region specialized for sperm storage, the seminal vesicle. At the post-vesicular region of each of the deferent ducts an accessory gland emerges. The seminal vesicle and the accessory gland are covered with a capsule forming a vesicle-gland complex, also observed in some species of North American *Polistes*. Sperm are released from testes in bundles, which are disorganized inside seminal vesicles. In the testicular follicles, 95 spermatozoa were observed per cyst on average.

## Introduction

The Vespidae family is currently classified into six subfamilies that are apparently monophyletic: Euparagiinae, Masarinae, Eumeninae, Stenogastrinae, Vespinae, and Polistinae (Carpenter 1991). The molecular phylogeny of Vespidae and the evolution of sociality in wasps supported closer phylogenetic relationships of Eumeninae to Polistinae + Vespinae than to Stenogastrinae, from which they concluded that social behavior has independently evolved twice in the wasp family Vespidae (Schmitz and Moritz 1998). Analysis of the realigned sequences also supports monophyly of Vespidae, as well as monophyly of social wasps, with the Stenogastrinae being more closely related to Polistinae + Vespinae than are Eumeninae (Carpenter 2003).

In the subfamily Polistinae, *Polistes* has about 200 species distributed throughout the world mostly in the tropical region (Richards 1978; Gauld and Hanson 1995). This genus has been widely studied and is considered the “key genus” for understanding evolution of the social insects and social behavior among wasps (Evans 1959; Schmitz and Moritz 1998; Carpenter 2003).

The reproductive system in males of Hymenoptera demonstrates considerable morphological differences among the species. Such differences may be related to the presence or absence and size or shape of structures, as well as its position along the reproductive tract (Dirks and Sternburg 1972; Dallacqua and Cruz-Landim 2003; Ferreira et al. 2004; Araújo et al. 2005a; Bushrow et al. 2006; Moreira et al. 2008). Sperm morphology has revealed a considerable number of features that differ among taxa (Araújo et al. 2005b; Brito et al. 2005; Zama et al. 2007; Lino-Neto et al. 2008a; Mancini et al. 2006, 2008), indicating another possible source of characters that may contribute to understanding the systematics of these insects.

In insects, germ cell development of males generally occur in compartments, called cysts. These cysts are found inside tubules or testicular follicles and are formed by clones of germ cells surrounded by a layer of non-germ epithelial cells (Baccetti and Bairati 1964). During spermatogenesis, the spermatogonia undergo mitotic divisions that generate a constant number of cells in each cyst. After two meiotic divisions, these cells become spermatids (Lindsley and Tokuyasu 1980; Oguma and Kurokawa 1984; Cruz-Landim 2001; Lino-Neto et al. 2008b). Each species has a particular number of spermatids inside the cyst, which is expressed as 2n, where “n” is usually equal to 5, 6, 7 or 8.

The number of spermatids/spermatozoa per cyst, which is determined by the number of cell divisions, is constant for each species, but may vary from species to species. Thus, this number has been used as additional information in the systematics of Hymenoptera (Schiff et al. 2001; Zama et al. 2007; Lino-Neto et al. 2008a).

Variation in the morphology of the reproductive system in males of Polistes was observed between European (Bordas 1895) and North American species (Dirks and Sternburg 1972). In the present work, the morphology of the male reproductive system, the spermatozoa and the number of spermatozoa per cyst were described for *Polistes versicolor versicolor* Oliver (Vespidae: Polistini). The aim is to contribute to knowledge of the male reproductive biology and also to reveal characters that may be useful for future studies in taxonomy and phylogeny of Hymenoptera, especially within Aculeata.

## Materials and Methods

Twelve adult males of *P. v. versicolor* were obtained from nests sampled on a farm in the municipality of Conceição do Castelo in the state of Espírito Santo, Brazil.

### Light microscopy

For the histological analysis, the reproductive systems of six males were fixed for 12 h in 2.5% glutaraldehyde in 0.1 M sodium cacodylate buffer, pH 7.2, and post-fixed in 1% osmium tetroxide. The material was dehydrated in increasing alcohol concentrations and embedded in Historesin® (GMA, Leica, www.leica-microsystems.com). Semithin sections were stained with 1% sodium toluidine borate and mounted in Entelan® (Merck, www.merck.com). The analysis and photographic records were made with an Olympus BX60 (www.olympus.com) microscope.

Suspensions of spermatozoa extracted from the seminal vesicles of six males were spread on clean glass microscope slides, which were fixed for 20 minutes in a solution of 4% (w/v) paraformaldehyde in 0.1 M sodium phosphate buffer, pH 7.2. After drying at room temperature, 100 randomly observed spermatozoa were photographed in a photomicroscope (Olympus, BX60) equipped with phase contrast.

For nuclear measurements, six slides of different males were stained for 15 minutes with 0.2 µg/ml 4,6-diamino-2-phenylindole (DAPI) in PBS, washed and mounted in 50% sucrose. They were then examined using an epifluorescence microscope (Olympus, BX60) equipped with a BP 360–370 nm excitation filter, and 100 nuclei were randomly photographed. All the measures were obtained with the Image Pro-Plus® software, version 4.5 (Media Cybernetics Inc.,www.mediacy.com), and the lengths were related to the total number of spermatozoa analyzed.

## Results

**Figure 1.**
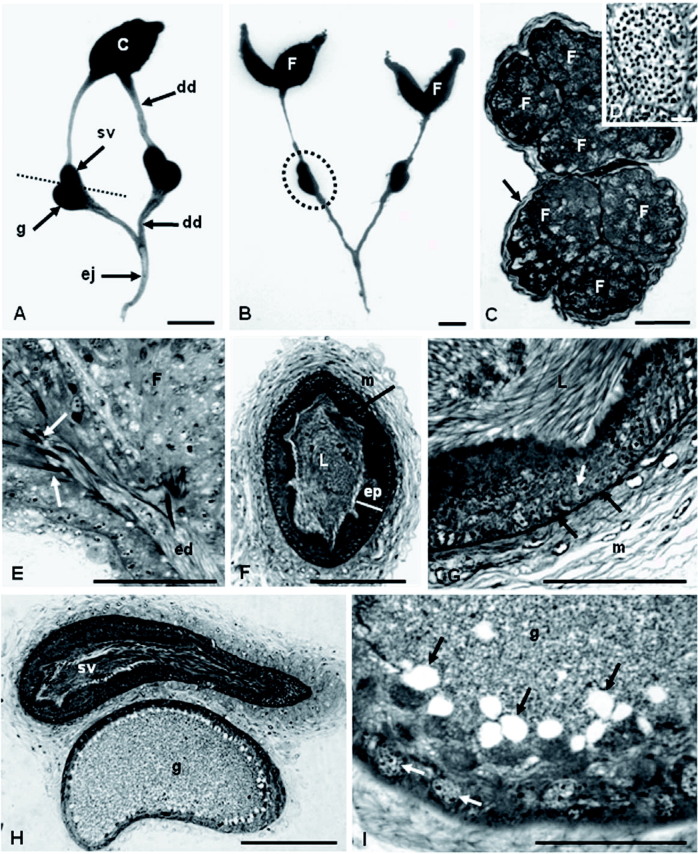
Photomicrograph of the anatomy (A–B) and histology (C–I) of the reproductive system of male *Polistes vesicolor*. **A.** Reproductive system showing the testes (T), seminal vesicles (sv) and accessory glands (g) separated by the broken line, deferent duct (dd) and the ejaculatory duct and **B.** showing the follicles (F) and the vesicle-gland complex involved by a single capsule (circle of broken lines). **C.** Transverse section of the testes showing six testicular follicles (F) and the testicular capsule (arrow). **D.** Inset: a cyst with 95 spermatozoa. **E.** Longitudinal section of a follicle (F), where the bundle of spermatozoa (arrow) is observed being released from the follicles (F) to the efferent duct (ed). **F.** Transverse section of the seminal vesicle showing the thick muscular layer (m), the epithelium (ep) and the lumen (L) with spermatozoa. **G.** Detail of the seminal vesicle's wall showing the epithelium comprising prismatic cells with spherical and basal nuclei (white arrow) and at the apical third, some vesicular inclusions (circle of broken lines); the epithelium is separated from the external muscular layer (m) by a thick basal membrane (black arrow). **H.** Longitudinal section of the accessory gland (g) and seminal vesicle (sv). **I.** Transverse section of the accessory gland completely filled with secretions (black arrow). Note the epithelium with basal nuclei (white arrow). Bars: A and B = 500 µm; C, F and I = 150 µm; D = 5 µm; E, G, H and J = 50 µm. High quality figures are available online.

The male reproductive system of *P. v. versicolor* consists of a pair of testes, each with three fusiform follicles (Figures 1A–C). The three follicles are covered by a single capsule and are entirely filled with cysts (Figure 1C). Each cyst has up to 95 spermatozoa on average (Figure 1D), indicating that, at least six mitotic cycles occur. During spermatogenesis, the cysts begin migrating along the follicles while they continue to differentiate. When the spermatozoa are completely mature, the cysts break open. The released bundles of spermatozoa (spermatodesmata) remain together, held by extracellular material that surrounds the anterior portion of their heads (Figure 1E). The spermatodesmata migrate to the efferent duct (Figure 1E), pass into the deferent duct, and are transferred to the seminal vesicles, where they are disorganized (Figures 1F–H).

The middle portion of the deferent duct is enlarged and presents a modified epithelium being transformed into a seminal vesicle (Figure 1F). The accessory glands connect to the beginning of the post-vesicular deferent ducts. The seminal vesicle and the accessory gland are surrounded by a single layer of conjunctive tissue or capsule, forming the vesicle-accessory gland complex (Figures 1A–B).

The epithelium of the seminal vesicle consists of prismatic cells with spherical, basal nuclei. In the apical third of these cells, some vesicular inclusions (Figure 1G) can be observed. In mature males, the vesicular lumen is completely filled with spermatozoa. The epithelium is separated from the external muscular layer by a thick basal membrane (Figures 1F–G).

**Figure 2.**
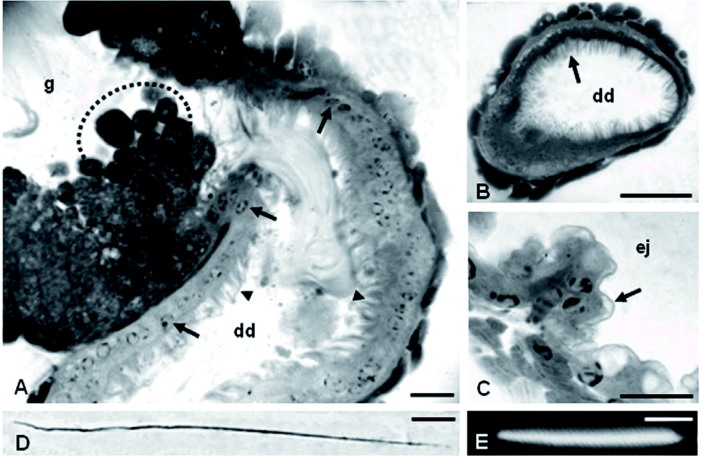
**A–C.** Histology of the male reproductive system of *Polistes vesicolor*. **D–E.** Photomicrograph of the spermatozoa and of the nucleus stained with DAPI, respectively. **A.** Longitudinal section from the region of the insertions of the deferent ducts (dd) into the accessory gland (g). Note the difference between the epithelium of the deferent duct (dd), showing basal and spherical nuclei (arrow), and the striated border (arrowhead) and the epithelium of the accessory gland (g) with several secretory vesicles at the apical portion (circle of broken lines). **B.** Transverse section of the deferent ducts (dd) showing the striated border (arrow). **C.** Transverse section of the ejaculatory duct (ej), showing the cuticle (arrow). **D.** Photomicrograph of the spermatozoon. **E.** Head region, stained with DAPI. Bars: A and D = 10 µm; B and C = 25 µm; E = 5 µm. High quality figures are available online.

The accessory glands are oval (Figures 1H–I), and their epithelium consists of prismatic cells with spherical and basal nuclei and large secretory vesicles in the apical cell portion (Figures 1I and 2A). The lumen is filled with granular secretions (Figures 1H–I).

The deferent duct epithelium is formed by cubical cells with a fairly evident striated border (Figures 2A–B). The deferent duct opens into the ejaculatory duct, whose epithelium, consisting of cubical cells, is completely covered by a thin cuticle (Figure 2C).

The spermatozoa of *P. v. vesicolor* measure about 110 µm in length (Figure 2D), and the nucleus is about 17 µm in length (Figure 2E).

## Discussion

Variation in the morphology of the reproductive system in males of *Polistes* was observed between European (Bordas 1895) and North American species (Dirks and Sternburg 1972). In general, the reproductive system of *P. versicolor* (species sampled in South America) is morphologically similar to that described for the North American species, *P. metricus* (Say), *P. exclamons* and *P. annularis* (Dirks and Sternburg 1972) as well as the European species *P. gallicus* (Bordas 1895). However, the vesicle-accessory gland complex observed in *P. versicolor* was described only for the North American species (Dirks and Sternburg 1972). These authors suggest that the geographical isolation of ancestral forms could have originated the anatomical pattern found in North American species. Our results point out that *P. versicolor* and the species of North American *Polistes* likely share a more recent ancestry when compared to the European species *P. gallicus*.

Although the reproductive systems in the different *Polistes* species are very similar, they differ markedly from those observed in *Ancistrocerus antilope* (Eumeninae) (Bushrow et al. 2006). In *A. antilope,* a single capsule involving the testicles and the seminal vesicles was reported — a pattern also observed in several species of bees of the subfamily Colletinae, Megachilinae, and Apinae (Ferreira et al. 2004). Many species of bees described by Ferreira et al. (2004) have shown conspicuous variation in the reproductive system of males, even within a single family.

In *P. v. versicolor*, the presence of an average of 95 spermatozoa per cyst indicates that there are, at least, six mitotic cycles in the spermatogonial proliferation phase. This number was observed in other species of Polistinae, such *Mischocyttarus* sp. (Brito et al. 2005) and *Agelaia vicina* (personal observation). In the family Sphecidae, six mitotic cycles were also observed in Sceliphrinae *Sceliphron fistularium* (Zama et al. 2005). In *Eumenes* sp. (Eumeninae), there are five mitotic cycles (personal observation). In the wasps of the family Crabronidae, there are also five mitotic cycles in Pemphredoninae (*Microstigmus*) (Zama et al. 2007). There are four cycles in Crabroninae *Trypoxylon* (Moreira et al. 2008) and in Sphecinae *Isodontia fuscipennis* (Zama et al. 2007). Thus, at least in Hymenoptera, the number of mitotic events during the spermatogonial proliferation phase is consistent within subfamilies and genera, but not in taxa above these categories.

The spermatozoa bundle released from the testes to the seminal vesicles, observed in *P. v.* *versicolor,* is a phenomenon common to Hymenoptera in general (Quicke et al. 1992; Moreira et al. 2004; Lino-Neto et al. 2008a). The observation of only mature cells at the final stage of the spermiogenesis and spermatozoa in the testes of adult *P. v. versicolor*, as well as in other species of *Polistes* (Dirks and Sternburg 1972) and in *A. antilope* (Bushrow et al. 2006), indicates that in these insects spermatogenesis begins in the pupal stage and that they produce spermatozoa only once. This has been observed in ants (Ball and Vison 1984) and bees (Dallacqua and Cruz-Landim 2003; Araújo et al. 2005a) is therefore common among the social Hymenoptera. In these species, the process of testis degeneration begins after the migration of the spermatozoa to the seminal vesicles. The same process possibly happens with social wasps. However, it was not observed in *P. v. versicolor* because the individuals were obtained from the nests and were, therefore, not yet sexually mature. The continuous production of spermatozoa is commonly observed in species that mate throughout the entire adult phase (Brockmann 1992; Garcia and Adis 1995; Coville et al. 2000; Buschini 2007; Moreira et al. 2008). This phase is longer for such species than for those that produce sperm only once.

As in most insects, the ejaculatory duct of *P. versicolor* is single, median and presents a cuticle, demonstrating its ectodermic origin. In *A. antilope* (Vespidae), the presence of two ejaculatory ducts was verified. They begin at the base of the accessory glands and later join to form the common ejaculatory duct (Bushrow et al. 2006). However, because these authors did not mention the presence or absence of a cuticle, it is not clear whether the ducts consist entirely of an ejaculatory duct or if they correspond to what we call the posterior region of the deferent ducts, followed by the ejaculatory duct.

In Hymenoptera, it has been observed that the morphology of the spermatozoa varies even among very closely related species. The spermatozoon of *P. v. versicolor*, as in the majority of the insects, is linear and slender. However, such cells are spiralled in some parasitic wasps, as in Chalcidoidea and Platygastroidea (Lino-Neto et al. 2000; LinoNeto and Dolder 2001). The spermatozoa length of *P. versicolor* (110 µm) is within the very wide range in size observed for Vespidae, which may vary from 13 to 577 µm (Quicke et al. 1992; Bushrow et al. 2006; Mancini et al. 2006; 2008).

This study supports the use of anatomical differences of the male reproductive system as a tool for the phylogenetic analysis among families of Hymenoptera or higher taxa. The number of spermatozoa per cyst may be used only when compared within the levels subfamily or genus. Nevertheless, the length of the spermatozoa may be helpful in taxonomic studies.
